# Violet Invader: Pediatric Soft Tissue Infection Caused by Chromobacterium violaceum

**DOI:** 10.7759/cureus.94555

**Published:** 2025-10-14

**Authors:** Zobiakhlui Chhakchhuak, B Lalrinpuia, George Lalthanmawia, TBC Ramengmawii

**Affiliations:** 1 Microbiology, Ebenezer Medical Center, Aizawl, IND; 2 Pediatrics, Ebenezer Medical Center, Aizawl, IND

**Keywords:** chromobacterium violaceum, gram-negative bacteremia, pediatric, sepsis, soft tissue infection

## Abstract

*Chromobacterium violaceum* is a Gram-negative bacillus known to be a rare cause of human infection. The organism enters the body through skin injury and usually presents as a localized skin infection that can progress to multiple abscesses and multiorgan failure. To our knowledge, this is the first reported case of skin and soft tissue infection caused by *C. violaceum* in the northeastern state of India, Mizoram. The organism was isolated from a pus sample obtained from a septic wound located on the foot and thigh of a pediatric patient. Though considered rare, this case report indicates that *C. violaceum* can be a potential cause of infection.

## Introduction

*Chromobacterium violaceum* is a Gram-negative bacillus, a facultative anaerobe, and oxidase-positive, belonging to the family Neisseriaceae. It is a common inhabitant of soil and water, causing opportunistic infections in humans by gaining entry through disrupted skin barriers. Infections caused by *C. violaceum* are rare, with fewer than 200 cases reported. They can range from localized skin and soft tissue infections and organ abscesses to bloodstream infections that may lead to sepsis. This organism can affect both adults and children and is associated with a high mortality rate. Reporting of infections by *C. violaceum* should be carried out with caution, taking into account geographical location and the local epidemiological profile, with most cases occurring in tropical and subtropical regions [1,2]. Given that most infections occur following skin trauma, we present a case involving a pediatric patient with a skin and soft tissue infection. Due to its high fatality rate and limited number of documented cases, we aim to highlight the virulent nature of an uncommon pathogen, providing insights for both clinicians and microbiologists. Additionally, we aim to emphasize the importance of prompt microbiological culture and sensitivity testing to guide appropriate antibiotic therapy and prevent the development of antibiotic resistance.

## Case presentation

**Figure 1:**
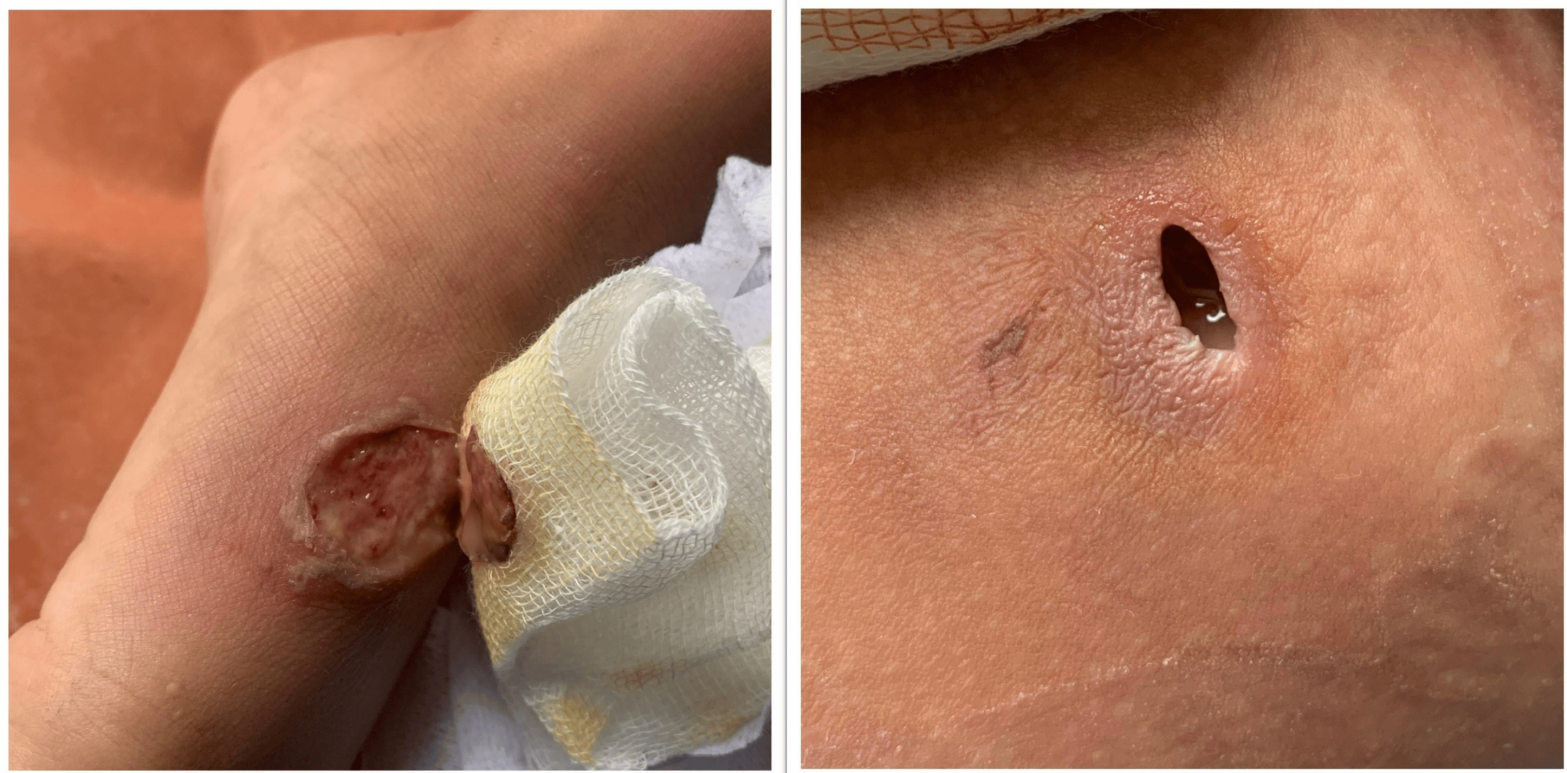
Lesion on the left foot (left) and left thigh (right)

**Figure 2:**
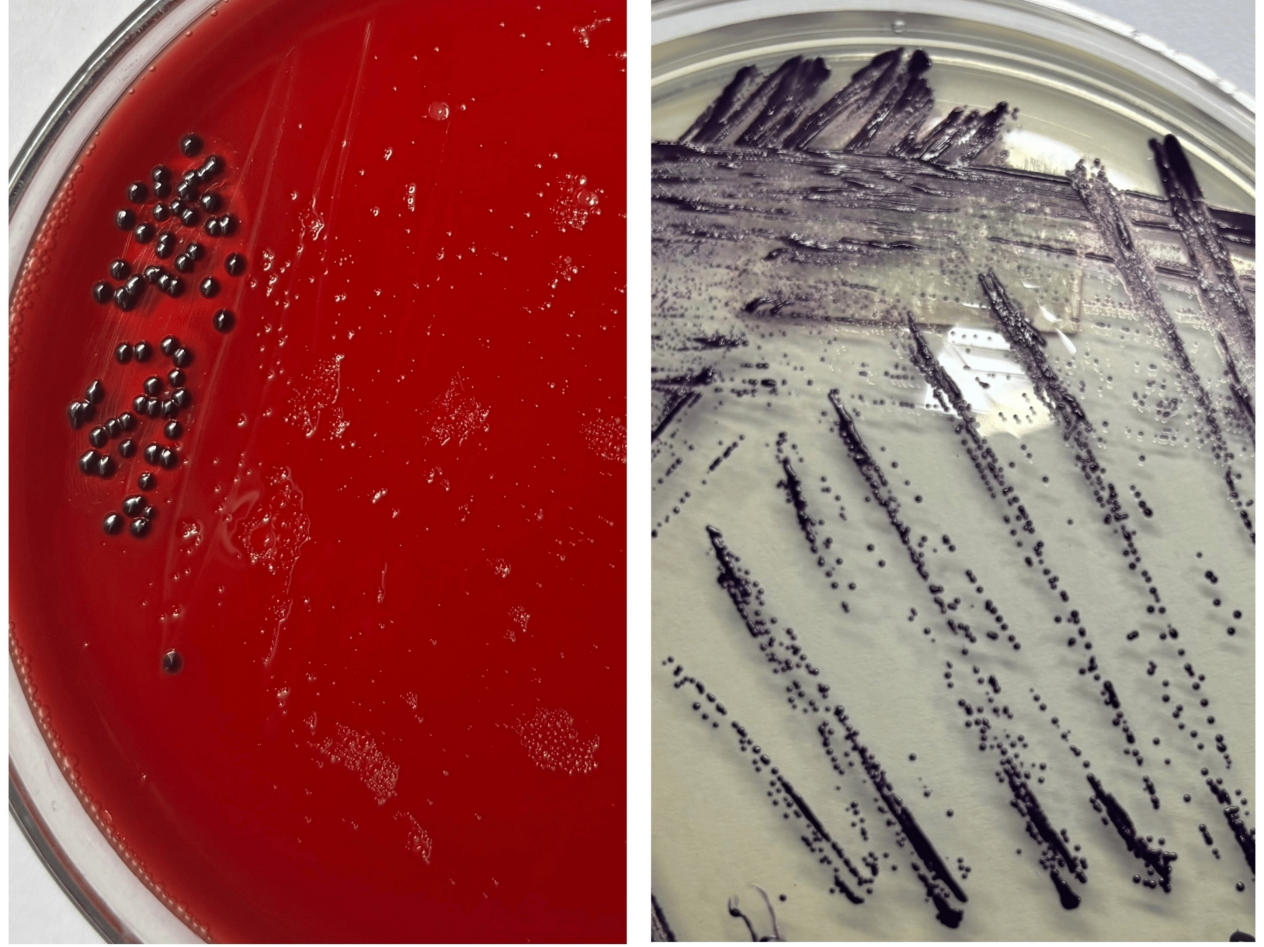
Violet-colored colonies of Chromobacterium violaceum on blood agar (left) and nutrient agar (right)

A seven-year-old boy came to the Emergency Department with complaints of fever for three days. He had a small wound on his left foot prior to the onset of fever. The child had reportedly sustained the injury while playing outdoors. The wound was managed conservatively at home by the parents with Betadine ointment before presenting to the hospital. On examination, the wound on the left foot had minimal pus discharge. It had initially started as a small pustular lesion that gradually increased in size. There was also a small abscess formation on the left thigh (Figure 1). His blood reports on admission were within the normal reference range except for markedly raised total leukocyte count (TLC) and C-reactive protein (CRP). Other tropical causes of fever, like scrub typhus IgM, dengue NS1 and IgM antibody, and TyphiDot IgM, were all ruled out by negative rapid immunochromatographic test (ICT) (Tables 1, 2). No significant abnormality was observed on the ultrasound of the whole abdomen. The patient was admitted on account of acute febrile illnesses under evaluation, with suspected suppurative lymphadenopathy. He was empirically started on intravenous ceftriaxone 500 mg given every 12 hours. Mupirocin ointment was also applied locally to the wound. The patient was subsequently referred to the Department of Surgery for incision and drainage of the abscess. Pus swab from the foot and aspirated pus from the thigh were sent to the Microbiology Department for culture and sensitivity testing on day 3 (D3) of admission. Plating was performed on blood agar and MacConkey agar, which were incubated at 37°C overnight. Gram staining revealed the presence of a few pus cells, and the culture plates showed heavily pigmented violet-colored, smooth, round, concave colonies on both agar media. The colonies were further subcultured onto nutrient agar for better visualization of the pigment production (Figure 2). Based on the distinct dark violet-colored pigmentation, *C. violaceum* was suspected to be the isolated organism. As the patient continued to have high-grade fever, the treatment regimen was changed to intravenous piperacillin-tazobactam 1.8 g and clindamycin 270 mg, administered every eight hours. Identification of the organism was further confirmed using the automated VITEK® 2 Compact system (bioMérieux, Inc.) using the Gram-negative identification card. Antimicrobial susceptibility testing revealed the organism to be susceptible to amikacin (MIC 16 µg/mL), ciprofloxacin (MIC ≤0.06 µg/mL), and levofloxacin (MIC ≤0.12 µg/mL). The organism was resistant to piperacillin-tazobactam, ceftazidime, cefoperazone-sulbactam, cefepime, aztreonam, and carbapenems. Based on the antibiotic report, the antibiotic was switched to intravenous ciprofloxacin 500 mg every 12 hours on day 5 (D5). The blood culture sent on admission was sterile after 48 hours and five days of incubation. 

**Table 1:** Hematological investigations of the patient TLC: total leukocyte count

Parameters	Results	Reference value
Hemoglobin	13.6 gm/dL	12-16.5 gm/dL
TLC	29600/mm³	4000-11000/mm³
Differential leukocyte count		
Neutrophils	95%	40%-80%
Lymphocytes	2%	20%-40%
Monocytes	3%	2%-8%
Total platelet count	3.48 lakh/mm³	(1.4-4.0) lakh/mm³

**Table 2:** Relevant investigations and their results ICT: immunochromatographic test; CRP: C-reactive protein

Investigations	Results	Reference value
CRP	193.31 mg/L	<5 mg/L
Dengue NS1 antigen and anti-dengue IgM antibodies qualitative assay	0.01	<1.0 (Index): Negative
		>1.0 (Index): Positive
Dengue IgM antibody	0.5	<1.0 (Index): Negative
		>1.0 (Index): Positive
Scrub typhus (*Tsutsugamushi*) IgG, IgM, and IgA antibodies detected by rapid ICT	Negative	
Typhi-screen test for *Salmonella Typhi* IgM antibody using rapid ICT	Negative	

The patient began to show clinical improvement; however, a small lump was noted on the left thigh, just below the site of the previous lesion. Local examination revealed no signs of abscess formation, and the patient remained afebrile with no other systemic symptoms. He was on intravenous ciprofloxacin for 14 days and then discharged with instructions to continue oral ciprofloxacin for another 10 days. He was asked to follow up at the outpatient department (OPD) upon completion of the antibiotic course. At the follow-up post-discharge, the patient was clinically well and asymptomatic. The previously noted lump persisted but had regressed in size. He was advised to continue oral ciprofloxacin and was placed under close monitoring for any recurrence or complications. The patient attended regular follow-ups for nearly two months. The lesions had fully healed with the formation of granulation tissue, and the lump had completely resolved. The patient was advised to monitor for any signs or symptoms of recurrence.

## Discussion

*C. violaceum* was initially described as a tropical pathogen with relatively few documented cases of human infection. It is characterized by its distinctive, dark violet pigmentation on culture media. Among the limited number of reported human infections worldwide, a total of 18 pediatric cases were reported between 2015 and 2024. In children, the presumed source of infection was typically soil or water, with the organism entering the body through traumatic breaches in skin or mucosal barriers. This is often followed by regional lymphadenopathy, as observed in our case [2]. In our patient, the suspected portal of entry was a lesion on the foot, which was subsequently followed by the development of an abscess on the ipsilateral thigh. 

The first reported case of *C. violaceum* in India was seen in an 11-month-old neonate, followed by a second case in a two-year-old pediatric patient. Both succumbed to the infection, underscoring the organism’s potential for high fatality [3]. The isolate described in this study represents the first known reported case of *C. violaceum* in the northeastern state of India. Given its rarity, *C. violaceum* was initially considered an unlikely causative agent in our patient. However, its isolation from both the foot and thigh confirmed it as the causative pathogen.

Review of literature showed a wide range of reported mortality rates for *C. violaceum* infections, varying from approximately 18% to as high as 53% [2,4]. The diverse mortality rate may be attributed to several factors, including the presence of co-morbidities, age of the individual, severity of disease (localized versus disseminated), and timeliness and appropriateness of antimicrobial therapy. Once infection is established, *C. violaceum* can disseminate locally, resulting in multiple abscesses and progressing to sepsis. As evident in our case report, the inflammatory markers of CRP were markedly raised to 30-fold from their normal reference value, along with TLC. These results confirmed the diagnosis of bacterial infection, underscoring the virulence and severity of *C. violaceum* infection. Due to its reported high fatality, the timely initiation of effective antimicrobial treatment is critical to preventing disease progression. In the present case, the specimen for culture was not sent on the day of admission, resulting in a delay in identifying the causative organism and exposing the patient to unnecessary empirical antibiotics. Resistance to the initial empirical regimens, third-generation cephalosporin (ceftriaxone) and Piperacillin-tazobactam was observed in our isolated organism. This finding underscores the importance of strict culture-guided therapy to prevent the misuse and overuse of antibiotics. It highlights a broader concern where antibiotic resistance remains a significant global health threat, contributing to increased morbidity, prolonged hospital stays, and healthcare costs. This experience also emphasizes the need for closer coordination between clinicians and microbiologists, with greater vigilance in identifying infection sources. *C. violaceum* is particularly known to be resistant to several classes of antibiotics [5]. As there are no established antibiotic susceptibility breakpoints for *C. violaceum* in the current CLSI and EUCAST guidelines, antibiotic susceptibility testing was interpreted following the CLSI M100 35th edition for *Pseudomonas aeruginosa, *which is considered to provide clinically relevant interpretive criteria for *C. violaceum *[6]. When it comes to treatment, various literature have reported successful outcomes with ciprofloxacin, either as monotherapy or in combination with piperacillin-tazobactam or trimethoprim-sulfamethoxazole, guided by antibiotic susceptibility profile [4,5]. 

Based on the limited resources and high risk of relapse, a prolonged treatment duration of up to three months is generally recommended for *C. violaceum*. A prolonged antibiotic course typically involves an initial phase of parenteral therapy followed by oral maintenance therapy for a total duration of two to three months [7,8]. In contrast, treatment of shorter duration has also been documented, but it was more frequently associated with relapse. The recurrence of relapse is hypothesized to be due to persistence of microabscesses or septic foci that may not be fully eradicated by therapy. Nonetheless, few reports have noted successful infection control even with shorter treatment duration [9]. Our case lies concurrent with the latter, where a shorter duration of one-month therapy was met with successful resolution of the infection.

## Conclusions

*C. violaceum* is associated with high fatality and morbidity rates due to its potential for rapid progression and dissemination. However, with increasing awareness of its pathogenicity and advancements in early detection methods, these rates are expected to decline substantially. Emphasizing the importance of timely culture and antibiotic susceptibility testing is critical for effective source control and the initiation of appropriate antimicrobial therapy. Furthermore, preventing relapse through continued antibiotic treatment and close patient follow-up remains a key component in the successful management of* C. violaceum* infections.
